# Coronary artery mycotic aneurysm in a patient suffering from subacute endocarditis: a case report and literature review

**DOI:** 10.3389/fcvm.2023.1188946

**Published:** 2023-08-03

**Authors:** Reza Hali, Mohammadbagher Sharifkazemi, Ahmad Yaminisharif, Jamshid Bagheri, Narges Shahbazi

**Affiliations:** ^1^ Cardiology Department, Tehran Heart Center, Tehran University of Medical Sciences, Tehran, Iran; ^2^ Cardiology Department, Nemazee Hospital, Shiraz University of Medical Sciences, Shiraz, Iran; ^3^ Cardiovascular Surgery Department, Tehran Heart Center, Tehran University of Medical Sciences, Tehran, Iran; ^4^ Pathology Department, Tehran Heart Center, Tehran University of Medical Sciences, Tehran, Iran

**Keywords:** cardiac infection, mycotic coronary aneurysm, endocarditis, anti-bacterial agents, *Viridans Streptococci*

## Abstract

Although mycotic aneurysm is a known and important disease in the cerebrovascular system, especially the brain, there are scarce reports about coronary artery mycotic aneurysms (CAMA). CAMA can occur not only in the context of endocarditis but also as a rare adverse event of coronary artery stenting, which has been used more extensively in recent years. Accordingly, it is essential to pay greater attention to its associated presentations and clinical course. Considering the scant evidence available, reporting the disease course of each patient with CAMA can help increase the physician's knowledge about this condition, which is why we are reporting this case. A 42-year-old man with diabetes was referred to our center with embolic left cerebellar infarction 3 months earlier, as well as a 2-month history of feverishness before his referral. His blood culture was positive for *Viridans Streptococci*, and he had paraclinical signs of inflammation and two- and three-dimensional transthoracic and transesophageal echocardiography (2D & 3D TTE and TEE) signs of aortic and mitral valves' infective endocarditis with the destruction of the aortic valve, severe aortic and mitral regurgitation, severe pulmonary hypertension, and moderate biventricular systolic dysfunction. Regarding the obviously dilated left main coronary artery on TEE images, contrast-enhanced chest multidetector computed tomography was performed for better assessment of coronary arteries with suspicion of CAMA, which confirmed aneurysmal dilatation of the proximal left main coronary artery. The presence of bacteria was confirmed on staining the valvular tissue, resected during the surgical replacement of aortic and mitral valves. As the cardiac surgeon considered CAMA resection and coronary bypass grafting high risk for the patient, he received parenteral antibiotic therapy, for 6 weeks. At 1-year follow-up, he was doing well with no signs/symptoms of endocarditis and well-functioning mechanical prosthetic valves. This case shows the significance of considering CAMA in the setting of endocarditis, resistant to medical and/or surgical therapy or in patients with coronary aneurysm, simultaneous with active endocarditis. Therefore, more attention should be paid to this extravalvular complication of endocarditis, and its possibility should be considered and investigated in any patient presented with valvular endocarditis, especially involving the aortic valve.

## Introduction

A mycotic aneurysm refers to localized, irreversible vascular dilatations, because of the weakening and destruction of the vessel wall, caused by a bacterial, fungal, or viral infection. The term “mycotic” refers to the particular shape of the aneurysm in the first case described and not the underlying pathology. So, as the fungal infection is not the mere cause of this infection, “infected aneurysm” would be a more accurate term. Femoral, aorta, intracranial, and visceral arteries are the more commonly involved vessels correspondingly (1).

Infected coronary artery aneurysm or coronary artery mycotic aneurysm (CAMA) is a very rare phenomenon, mainly observed in men, caused secondary to infective endocarditis (IE), percutaneous coronary artery intervention (PCI), and extracardiac infections, during which microembolization to the vasa vasorum and direct invasion of the pathogen to the arterial wall, or immune complex deposition results in vessel's wall injury and destruction (2). Although most CAMA cases are large (average 3.4 cm, max: 9 cm in diameter) at the time of diagnosis, non-specific clinical presentations, such as feverishness, malaise, and weight loss with leukocytosis, increased level of erythrocyte sedimentation rate (ESR) and C-reactive protein (CRP) makes CAMA diagnosis a challenge and includes it in the differential diagnosis of any “fever of unknown origin” (3). However, the presence of an infected aneurysm, especially with a cardiac/coronary origin, is rarely anticipated by such clinical presentations. Furthermore, most cases do not have any specific finding on the initial/routine clinical or paraclinical cardiac exam -such as cardiac auscultation or electrocardiogram-, so more accurate diagnostic methods, such as chest computed tomography (CT), are suggested for diagnosis (4).

Missed diagnosis of CAMA results in a high mortality rate because the aneurysm, especially those larger than 1–2 cm, will eventually grow and end in rupture or thrombosis frequently, even despite proper antibiotic therapy. Considering the acceptance of PCI as an appropriate therapeutic tool for several coronary diseases and its widespread use during the recent decades, it is anticipated that the incidence of CAMA has also increased (5, 6). However, few case reports available in the current literature make it difficult to estimate the rates of incidence and complications of CAMA or to determine the most accurate diagnostic tool. There is also scant evidence available in the literature in terms of the most appropriate therapeutic approach. Some experts have suggested medical treatment for smaller aneurysms and surgical treatment for larger ones (7); Even though it is (then again) not clear what precise cut-point should be considered for the surgical resection of the aneurysm or what setup should be considered. Accordingly, reporting the disease course and outcome of any patient with this condition can help increase the physician's knowledge and grow the available literature. Here, we report a case of CAMA in a 42-year-old man, who presented with a 2-month history of feverishness and was successfully diagnosed and managed at our center.

## Case presentation

A 42-year-old man with diabetes mellitus was referred with a 2-month history of feverishness and embolic ischemic left cerebellar infarction a month back. Serum tests revealed a positive blood culture for *Viridans Streptococci* organism with evidence of inflammation, including leucocytosis and increased levels of ESR and CRP. A brain CT scan without contrast was suggestive of a non-hemorrhagic stroke involving the left cerebellum (Supplementary Figure S1).

He had no past medical history of cardiovascular diseases and no first-degree family history of valvular or ischemic heart disease or genetic disorder. The psychological history of the patient was unremarkable.

**Figure 1:**
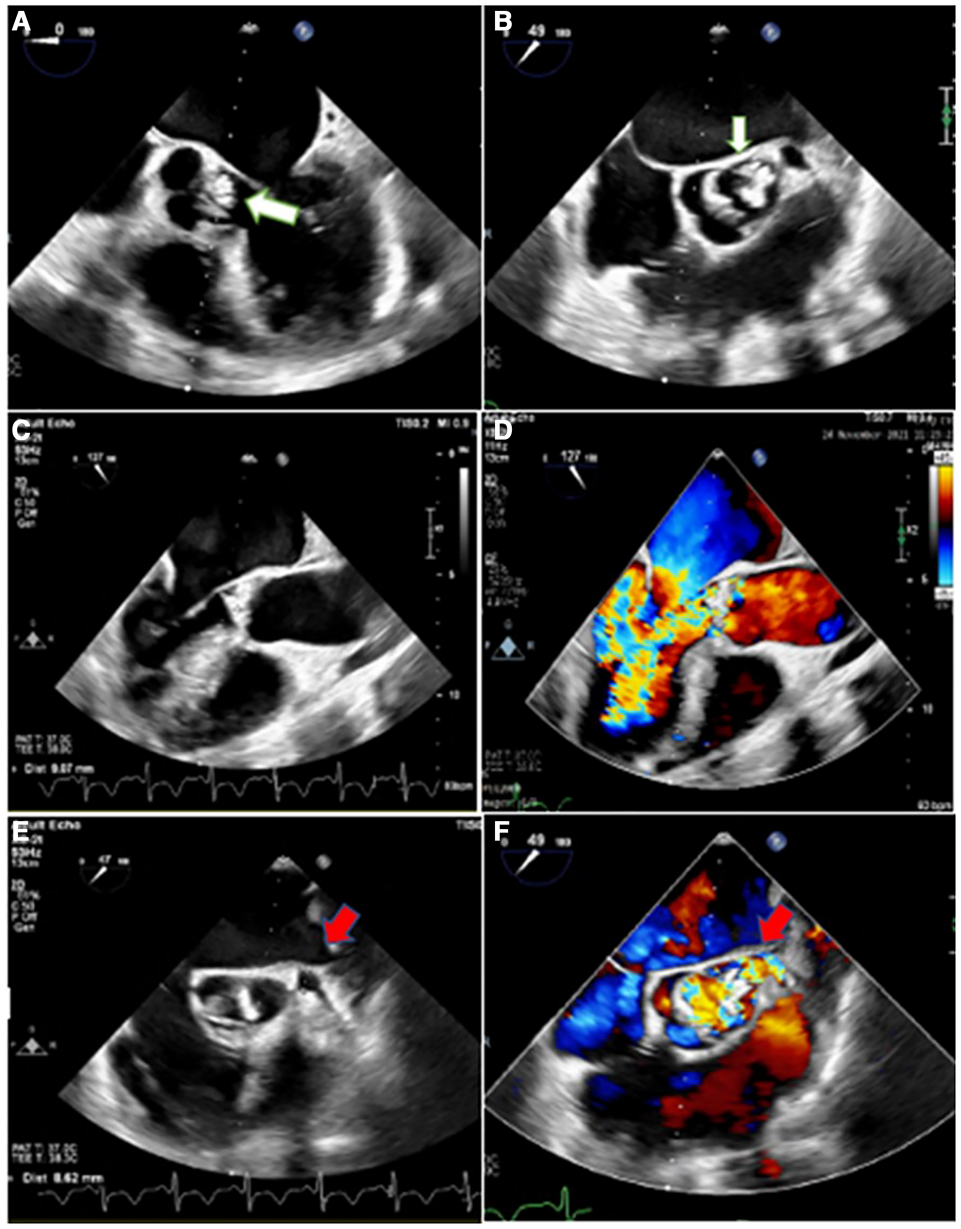
Two-dimensional transesophageal echocardiography in different views, illustrating irregularly thickened and destructed bicuspid aortic valve (non-coronary cusp and right coronary cusp fusion) with partially ruptured left coronary cusp (white arrow) and a few small mobile vegetative particles, suggestive of aortic valve involvement and destruction by infective endocarditis (**A,B**), heralding severe aortic insufficiency (**C,D**). Moreover, it shows the dilated left main coronary artery (red arrow) with turbulent blood flow inside (**E,F**).

**Figure 2:**
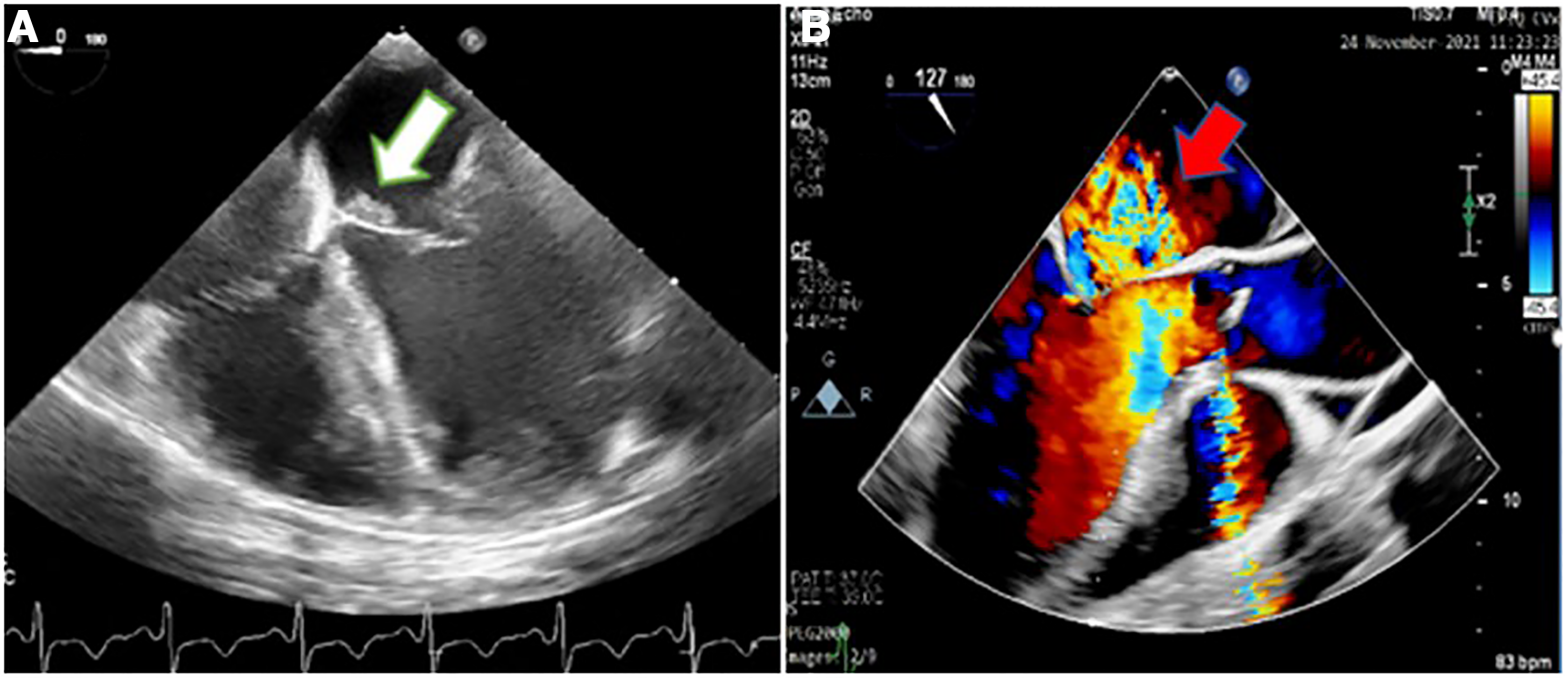
Two-dimensional transesophageal echocardiography showing an irregular thickening over the atrial side of the base of anterior mitral valve leaflet (white arrow) (**A**) suggestive of the involvement of the mitral valve by infective endocarditis, which caused severe mitral regurgitation, demonstrated in color Doppler flow study (red arrow) (**B**).

Considering the easily audible systolic and diastolic murmurs on cardiac auscultation, two- and three-dimensional transthoracic and transesophageal echocardiography (2D & 3D TTE and TEE) were performed for better assessment, which revealed an irregularly thickened and destructed bicuspid aortic valve (fusion of non-coronary and right coronary cusps), a few small mobile vegetative lesions, and a partially ruptured left coronary cusp of the aorta, resulting in severe aortic regurgitation (Figure 1). The patient had no aortic ectasia or dilation in view of bicuspid aortic valve or any evidence of endarteritis elsewhere. IE involvement of the mitral valve in the base of the anterior leaflet, adjacent to the aortic valve, with severe mitral regurgitation was also evident (Figure 2). A tricuspid regurgitation with moderate severity and severe pulmonary hypertension (60 mmHg), and severe left ventricular dilatation, accompanied by moderate biventricular systolic dysfunction were also detected. The left main coronary artery was dilated on 2D & 3D TEE.

**Figure 3:**
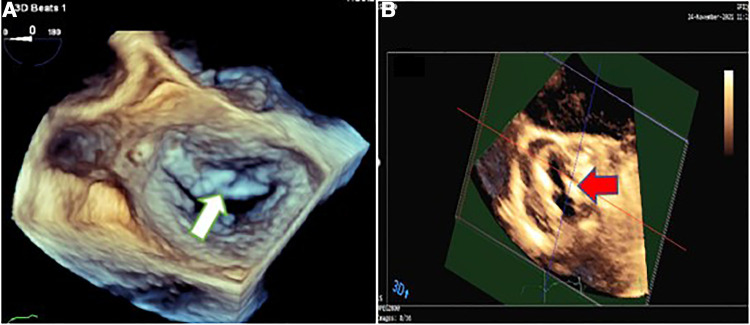
Three-dimensional transesophageal echocardiography showing mitral valve endocarditis with irregular thickening over the atrial side of the base of anterior mitral valve leaflet (white arrow) (**A**) and irregularly thickened and destructed bicuspid aortic valve involved by infective endocarditis (red arrow) (**B**).

**Figure 4:**
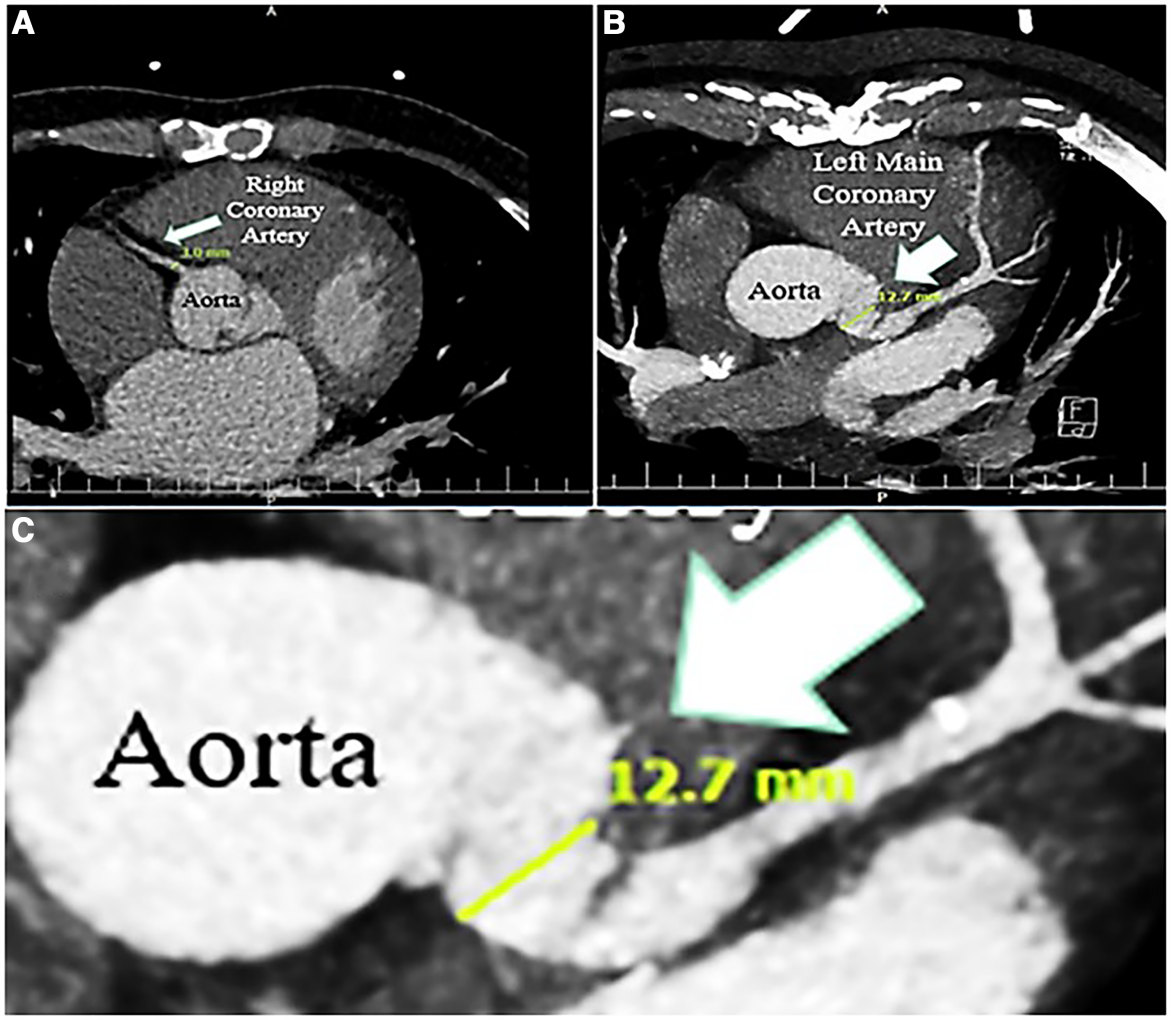
Contrast-enhanced chest computed tomography scan, showing normal caliber right coronary artery (3 mm) (**A**), and dilated left main coronary artery (12.7 mm); zoom out (**B**), and zoom in (**C**).

The vegetative lesions on both aortic and mitral valves raised the suspicion of a probable simultaneous coronary artery involvement by IE (Figures 2, 3). Contrast-enhanced chest multidetector CT scan performed showed aneurysmal dilatation of the left main coronary artery (12.7 mm diameter) with an irregular border, originating from the left coronary sinus extending to the proximal LAD artery, suggestive of CAMA (Figure 4). The algorithmic process of diagnosis in the presented case is provided in Supplementary Figure S2.

Surgical replacement of aortic and mitral valves was done after 2 weeks of intravenous antibiotic therapy. Control echocardiography (repeated after 2 weeks of therapy) showed no changes in echocardiographic findings. Preoperative assessments (sestamibi dipyridamole stress test) showed no signs of coronary artery disease, requiring concomitant coronary artery bypass grafting. During surgery, the aortic and mitral valves were replaced by proper mechanical bileaflet valves (St. Jude 21 and St. Jude 30 for aorta and mitral positions, respectively) and tissue samples were sent for pathological examination. Gross observation during the operation confirmed abnormal dilatation of the left main coronary artery, which extended to the proximal LAD, in favor of CAMA. The cardiac surgeon considered CAMA resection and coronary bypass grafting to be of high risk. Therefore, the surgeon ended the surgery with mere valvular replacement and long-term antibiotic therapy was selected as the treatment of CAMA in this patient.

Histopathological evaluation of the excised valves and surrounding tissues confirmed valvular destruction, inflammation, and infection with gram-positive cocci (Supplementary Figure S3). The patient was uneventful during postsurgical weaning, recovery, and admission. He was discharged after 6 weeks of combined parenteral and PO antibiotics, prescribed for 2 more weeks to complete a 2-month period of antibiotic therapy, consisting of a combination of rifampicin, Tavanex, and amikacin (selected based on the antibiogram results). At 1-year follow-up, he was doing well with well-functioning mechanical prosthetic valves on echocardiography and no change in size of coronary aneurysm on the contrast-enhanced chest multidetector CT scan (Supplementary Figure S4). For all diagnostic tests and selection of therapeutic procedures, the patient was informed about the risks and benefits and signed the written informed consent. After treatment, the patient was completely satisfied with the care provided.

## Discussion

CAMA is a serious clinical condition with the risk of rupture and myocardial infarction (8); short-term mortality rate of CAMA is reported at about 40%, confirming poor prognosis of this condition (9). Few cases have been reported, limited to a total of <100 cases until 2017 (9); thus, more studies are required to estimate the accurate incidence of CAMA.

In view of pathophysiology, the available evidence suggests IE as the source in most cases of CAMA (5, 6, 10), which predisposes the patients to other complications, such as hemorrhagic and embolic (cerebrovascular) events (11). Stent implantation in coronary arteries is also frequently reported as an important source of bacteremia and bacterial seeding, resulting in CAMA; but our patient had no history of previous coronary stenting and neither was immunocompromised to be further predisposed to any possible systemic infection as the cause of CAMA; therefore, his coronary infection can be attributed to endocarditis because of the bicuspid aortic valve and direct extravalvular extension of IE or possible bacterial seeding of the proximal coronary artery, secondary to valvular involvement by IE.

Another important concern about CAMA, based on its cause, is the high risk of comorbidities resulting from IE-related cardiac damage, such as valvular destruction (in cases with concomitant IE) (12). As in the case presented here, concomitant IE was a probable cause of CAMA, which could have been the source of destruction of aortic valves and involvement of the mitral valve with severe regurgitation. Fortunately, this case was diagnosed on time and successfully managed before the occurrence of any severe complications or rupture of the aneurysm. Unfortunately, there are some reports available of patient's expiry, even after therapeutic measurements, mainly because of the challenging management of coronary artery rupture (8, 13).

The dubious nature of CAMA makes well-timed diagnosis an important parameter in the proper and successful management of this potentially fatal condition. Although the clinical symptoms are mainly non-specific and general (3), some patients may present with acute symptoms, such as high fever, shortness of breath, chest pain, nausea, and vomiting, associated with coronary complications itself or concomitant cardiac conditions, like pericardial effusion (4), pericarditis (14, 15) or IE. Nevertheless, the case presented here did not have any acute or significant cardiac-related symptoms at referral, no evidence of coronary artery disease (based on the results of sestamibi dipyridamole stress test), and his mere clinical symptom was a 2-month history of feverishness with a history of recent brain ischemic embolic event, of course. As there are no clinical presentation or laboratory findings, specific to CAMA, it is important to exclude other diseases with similar presentations in clinical approach to the patients; differential diagnoses, such as reactivaltion of rheumatic fever and systemic lupus erythematosus, should be considered. Accordingly, in our case, we were able to exclude other diseases by relevant laboratory tests (16). However, the presence or source of CAMA was not predictable initially, based on clinical presentation only, in our patient, as he reported no history of any known cardiac disease or previous cardiac intervention and complained of no significant cardiac-related symptoms. In the meantime, the morphological characteristics of the coronary artery, observed on cardiac imaging, in addition to the presence of valvular involvement by IE and the suggestive paraclinical data, guided the physicians toward this diagnosis. Paraclinical data in our case showed evidence of bacteremia with *Viridans Streptococci*. *Staphylococcus aureus* and *Streptococcus viridans* have been previously described to be the two most common pathogens in CAMA (2, 17). Furthermore, our patient had high levels of inflammatory markers in the blood (leukocytosis, increased level of ERS and CRP), which are non-specific markers and were thus not helpful for diagnosis of CAMA, regarding concomitant valvular involvement with IE. So, the key diagnostic finding in our case was the unusual focal dilation of the coronary artery in the echocardiographic examination, confirmed by chest MDCT, besides valvular involvement by IE.

Cardiac imaging in CAMA can detect coronary dilation with occasional lobulated contour or saccular shape, wall thickening or mural thrombus, and also possible associated abnormalities in the pericardium or valves. We have identified the characteristics of the coronary aneurysm by contrast-enhanced chest CT scan. Some authors have used angiography for an accurate diagnosis (18), which we consider high risk for such patients, especially in cases with involvement of the very proximal portion of coronary arteries, near the ostial region; thus, we recommend the use of CT angiography as a better diagnostic tool, in case the physician requires angiographic parameters for accurate diagnosis (17). Magnetic resonance angiography, also, has several limitations, such as limited access, long examination time, and motion artifacts, and thus not preferred (3, 17). Some experts have also described the use of TEE for the diagnosis of CAMA (19). In our experience, TEE could successfully reveal the abnormalities of the proximal coronary artery and propose the probability of the presence of CAMA, in addition to valvular abnormalities. Considering the scant evidence available and the rarity of CAMA, it is not easy to suggest one imaging tool as the gold standard. More studies are required to compare the diagnostic accuracy of different imaging modalities and suggest one as the superior method (17).

Treatment of CAMA consists of antibiotic therapy, in addition to surgical resection or de-roofing and debridement of the infected aneurysm, in combination with arterial ligation, with or without distal bypass of the artery, and vascular reconstruction; in case of rupture, endovascular therapies may be required (9, 17). The size of aneurysm, extent, and location of the coronary artery involved, morphological appearance and characteristics of the aneurysm, clinical status, and other factors may also be important for proper decision-making and therapeutic approach for each patient; although these parameters have not been defined well in literature. Other treatments, like covered stent, which can be used in other types of coronary aneurysms, are also not an appropriate choice in CAMA, considering the infection background (17, 20); apparently, long-term antibiotic therapy is the treatment of choice in CAMA. However, there is still no approved evidence-based guideline available for the treatment of CAMA, and the best therapeutic option is not defined; hitherto, it is certain that CAMA requires clinical vigilance and great attention to be diagnosed and managed appropriately.

Fortunately, our patient did not develop coronary rupture or other complications by the time he was diagnosed, and he was successfully managed by a hybrid treatment strategy (combination of surgery for treatment of valvular IE and medical therapy for CAMA), had an uneventful postoperative period, no clinical symptoms, and negative paraclinical results (no evidence of IE of the prosthetic valve) until the last follow-up (1 year); the favorable prognosis of our patient could be related to the relatively small size of the aneurysm and limited involvement of coronary artery with no visual signs of the high-risk aneurysm (which predispose the patients to complications or rupture) in preoperative imaging modalities, and gross visual observation by the surgeon at the time of surgery. For the choice between surgery and medical treatment (no surgery), in this case, the surgeon suggested a no-surgery strategy (antibiotic therapy), considering the high risk of surgical resection. Antibiogram-driven antibiotic therapy was also prescribed, and 1-year follow-up showed favorable results, which indicated that the treatment strategy applied could be appropriate in carefully selected patients.

In conclusion, the case presented here had several notes to be kept in mind. First of all, the symptoms of CAMA are non-specific, thus, diagnosis can be confounding and needs a high level of suspicion, as mycotic aneurysms are an infrequent type of aneurysms, and the coronary artery is a rare site of this condition. As CAMA has a poor prognosis and high risk of rupture, myocardial infarction, and other cardiac complications, special attention should be paid for considering CAMA in differential diagnosis, when the patient has coronary aneurysm, simultaneous with active endocarditis or in patients with endocarditis, resistant to medical and/or surgical therapy. Blood tests are helpful in determining bacteremia and inflammation. Focused imaging approaches should be considered, especially if the case is accompanied by other more obvious sources of infection (like the valvular endocarditis in our case), which makes the diagnosis doubtful. The etiology of CAMA is mainly attributed to IE or previous cardiac vascular interventions, especially in patients without immunosuppressive conditions. But, the possibility of concomitant CAMA, in the absence of these conditions, should be also kept in mind and greater attempt should be applied for detection of other sources of infection in cardiac structure. In our patient, the bicuspid aortic valve was the most probable cause of valvular IE, resulting in coronary aneurysm. The choice of treatment, antibiotic therapy and/or urgent surgical intervention, should be made based on patient's conditions; surgery is recommended in cases with large or high-risk aneurysms.

There are few case reports available in the literature about CAMA; however, we believe that the true incidence is much higher than that reported, considering the high probability of missed diagnosis. This notion has to be confirmed by further epidemiological studies.

## Data Availability

The raw data supporting the conclusions of this article will be made available by the authors, without undue reservation.

## Supplementary material

SUPPLEMENTARY FIGURE S1

Brain computed tomogram without contrast, showing a low-density region in the left cerebellum, suggestive of non-hemorrhagic stroke.

SUPPLEMENTARY FIGURE S2

The algorithmic process of diagnosis in the presented case.

SUPPLEMENTARY FIGURE S3

The pathological specimen indicates destruction of valvular structure with overlying fibrin thrombus and mild focal infiltration of acute inflammatory cells in the setting of previous antibiotic treatment, mitral valve (H&E staining 10X objective) (A). Effacement of the valvular structure due to severe inflammation, and infiltration of acute inflammatory cells is evident, aortic valve (H&E staining 10X objective) (B). Rare gram-positive cocci (black arrowhead). (Gram staining 100X objective) (C).

SUPPLEMENTARY FIGURE S4

The results of contrast-enhanced computed tomography, illustrating the diameter of the coronary aneurysm before cardiac surgery (A), and 1 year later (B).
