# Supplementary figures and images for: Coronary artery mycotic aneurysm in a patient suffering from subacute endocarditis: a case report and literature review
Source: Front Cardiovasc Med. 2023 Aug 3;10:1188946. doi: 10.3389/fcvm.2023.1188946 (PMC10435280; doi:10.3389/fcvm.2023.1188946)

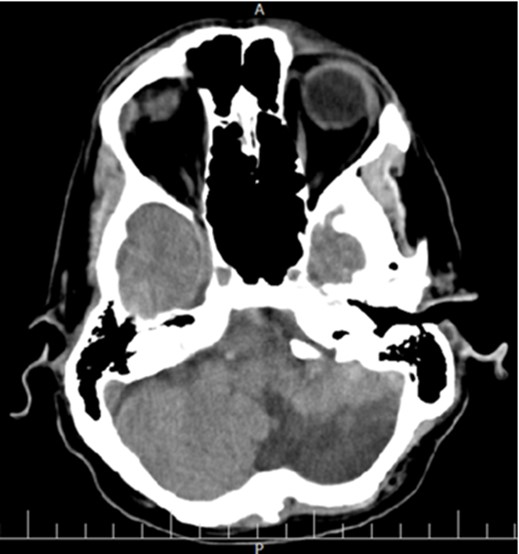

Supplement: Supplementary file 1 [file Image1.jpeg]

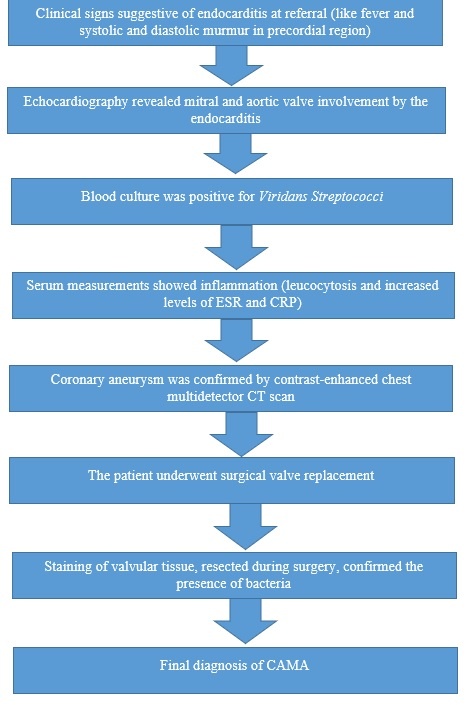

Supplement: Supplementary file 2 [file Image2.jpeg]

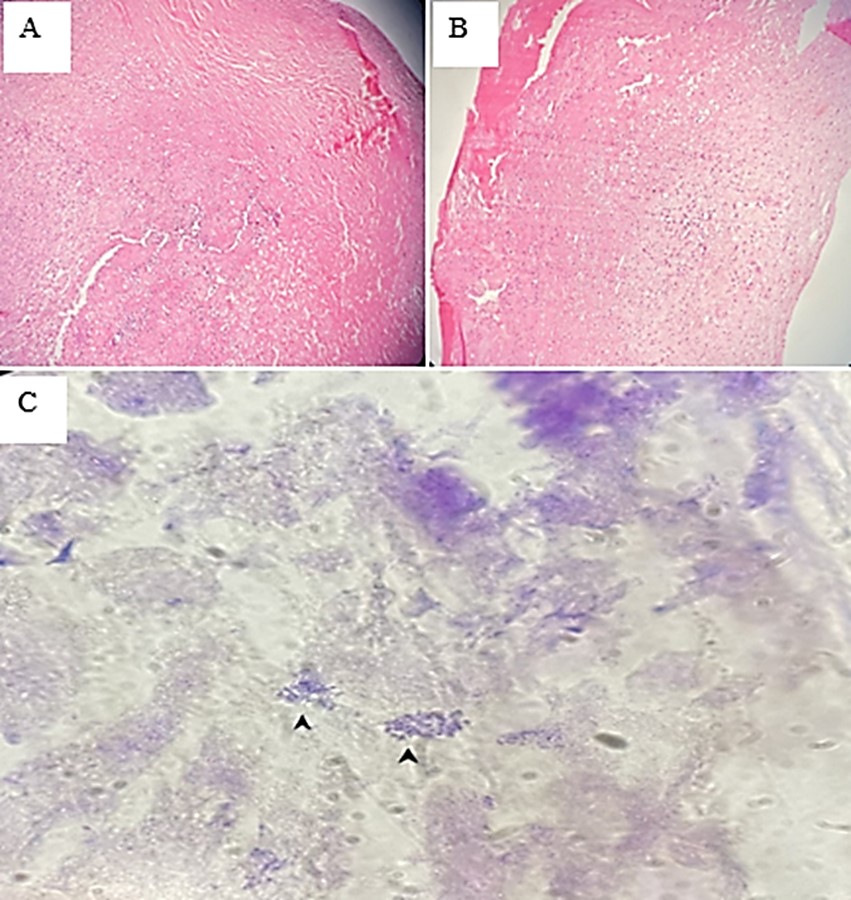

Supplement: Supplementary file 3 [file Image3.jpeg]

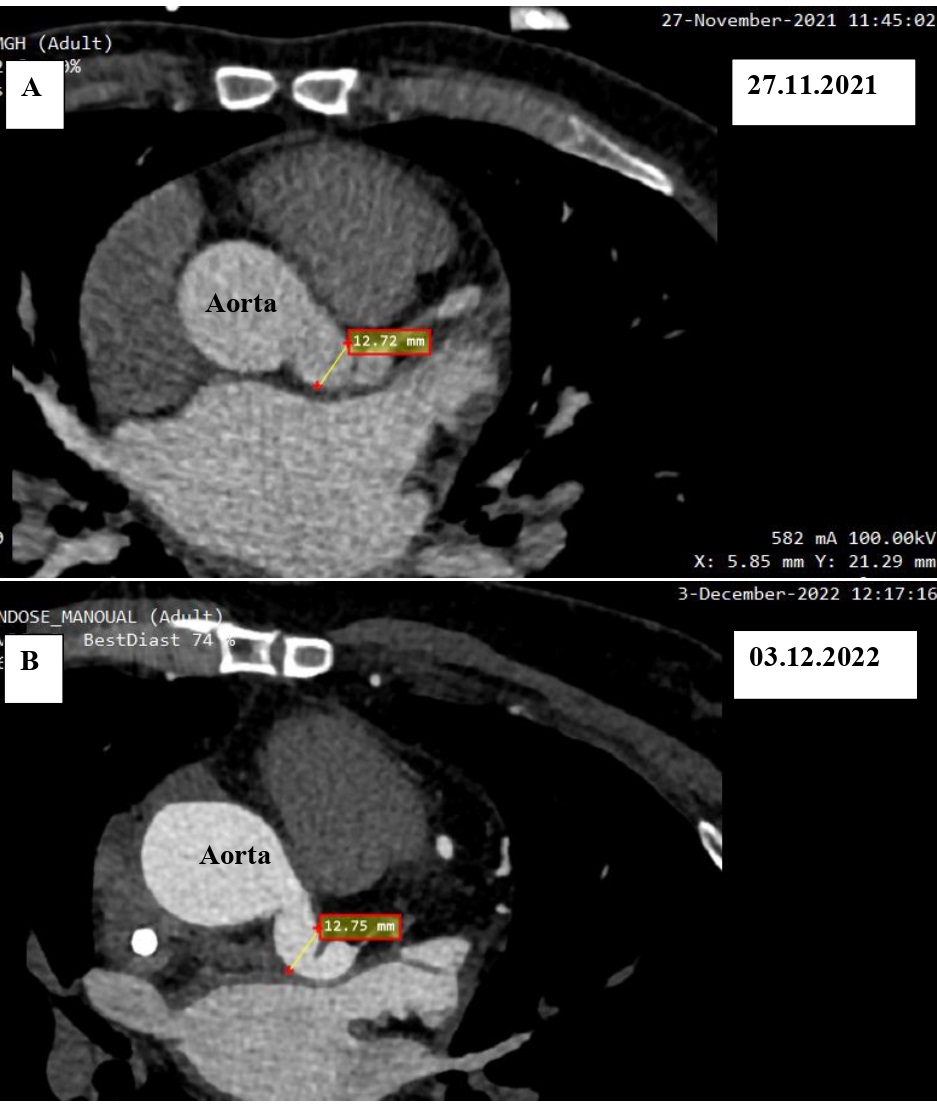

Supplement: Supplementary file 4 [file Image4.jpeg]
